# Physician counseling of young adults with rapid weight gain: a retrospective cohort study

**DOI:** 10.1186/1471-2296-11-31

**Published:** 2010-04-30

**Authors:** Joyce W Tang, Robert F Kushner, Jason Thompson, David W Baker

**Affiliations:** 1 Division of General Internal Medicine, Northwestern University Feinberg School of Medicine, Chicago, USA; 2 Institute for Healthcare Studies, Northwestern University Feinberg School of Medicine, Chicago, USA

## Abstract

**Background:**

The incidence of weight gain is highest during young adulthood. Our study aims to describe weight gain patterns among young adults and to evaluate physician recognition of and counseling for rapid weight gain.

**Methods:**

This retrospective cohort study included patients ages 18-35 at an academic internal medicine clinic between 2004-2008. We conducted chart reviews to determine weight change over time, whether weight gain greater than 3 lbs/year was documented, whether counseling was provided, and whether patients became overweight. We categorized weight gain documentation by location on the problem list, encounter diagnosis, or note text. We categorized counseling as weight-specific or general diet and exercise counseling. We used Chi-square tests to evaluate the relationship between weight change over time and the following variables: gender, diagnosis of weight gain, and counseling for weight gain. Fisher's Exact test was used to test for an association between diagnosis and counseling category.

**Results:**

The study included 365 patients. Weight gain was greater than 3 lbs/year for 24% (90/365) of patients, of whom 56 (15%) gained 3-5.9 lbs/year, and 34 (9%) gained more than 6 lbs/year. Among patients gaining more than 3 lbs/year, physicians documented weight gain as a problem in only 10% (9/90). Of the 9 patients for whom weight gain was documented, physicians provided weight-specific counseling in three, and general diet and exercise counseling in four. Of the 81 individuals with no documented diagnosis of weight gain, 63% had no documented counseling, but 34% received general diet and exercise counseling. Among patients with over 180 days of follow-up, 8% (10/126) became overweight.

**Conclusions:**

Physicians infrequently recognize or counsel for weight gain among young adult patients. Improving identification of patients with rapid weight gain can provide an opportunity for tailored weight-related counseling.

## Background

Epidemiologic studies report that the incidence of weight gain is greatest during young adulthood[1,2]. Normal weight individuals at age 30 have a 50% risk of becoming overweight and a 25% risk of becoming obese over 30 years[3]. Modest weight gain during adulthood has been associated with a higher risk of coronary heart disease, stroke, breast cancer[4-6] and possibly an increase in mortality[7]. Although normal weight patients rarely receive physician advice to maintain normal weight,[8]. the subset who are rapidly gaining weight constitute a higher risk group that may benefit from targeted counseling.

No prior studies, to our knowledge, have evaluated physician recognition of or counseling for weight gain. Our study aimed to describe weight gain patterns among normal weight young adults within a clinic setting and to evaluate physician documentation and counseling for weight gain.

## Methods

This was a retrospective cohort study. The study site was an academic general internal medicine clinic (Northwestern Medical Faculty Foundation General Internal Medicine clinic) located in Chicago, IL. The clinic serves predominantly an upper-middle class population. We queried the electronic medical record (EMR) to identify adults ages 18-30 at the time of their initial visit to the clinic between 2004-2006 and who were of normal body weight (Body Mass Index [BMI] 18.5-24.9 kg/m^2^) at all visits prior to and during a subsequent preventive visit. Preventive visits were defined as visits with ICD-9 codes corresponding to preventive visits (V70.0, V70.3, V70.5, V70.8, V72.31) or which were slotted to be 40 minutes in length. We excluded pregnant women and individuals with less than 180 days between their first documented weight and last preventive visit weight to prevent inflation of short-term weight variability. We excluded patients without a preventive visit because we anticipated that weight gain would not be routinely addressed during acute care visits. The Institutional Review Board of Northwestern University approved this research.

### Measures

Weight change over time (lbs/year) was calculated for each patient using the following formula:(1)

We categorized weight gain greater than 3 lbs/year as rapid weight gain in order to balance capturing significant weight gain (3 pounds approximates half a BMI unit) with a sufficiently low threshold to allow for early detection. We reviewed charts for all patients who gained greater than 3 lbs/year to determine whether weight gain was documented, whether counseling was provided, and whether the patient or physician initiated discussions about weight gain. Notes from all patient visits between 2004 and 2008 were reviewed. Documentation of weight gain was categorized by the following descending hierarchical levels: problem list, encounter diagnosis, note text, or no documentation. Diet and exercise counseling was categorized into three descending hierarchical levels using the following criteria: **weight-specific counseling **if physicians documented advice to lose or maintain weight or if diet or exercise counseling was documented in the context of noted weight gain; **general diet or exercise counseling **if physicians documented diet or exercise counseling in the context of general health maintenance or treatment of a medical condition or if physicians documented that patients had excellent health habits with no need for counseling; or **no documented counseling**.

Last weight class was determined for patients with at least 180 days between their last preventive visit and last available weight using last recorded weight. Weight class was categorized as normal weight (BMI 18.5-24.9 kg/m^2^), overweight (BMI 25-29.9 kg/m^2^), or obese (BMI>30 kg/m^2^).

### Statistical analysis

Analyses were conducted in 2009 using STATA 10.0 (College Station, TX). We used Chi-square tests to evaluate the relationship between weight change over time and the following variables: gender, diagnosis of weight gain, and counseling for weight gain. Fisher's Exact test was used to test for an association between diagnosis and counseling category.

## Results

The study sample included 365 patients; 75% were female and the average age was 25.6 ± 2.7 years at the first clinic visit. Ninety-seven percent of patients had private insurance. Co-morbidities in the patient sample included psychiatric diagnoses (64/365, 18%), hypertension (4/365, 1%) and diabetes (3/365, 1%). Patients were seen at a median of eight clinic visits. Weight change (over a median of 685 days) was greater than 3 lbs/year for 90 patients (24.3%), of whom 56 (15%) gained between 3-5.9 lbs/year, and 34 (9.3%) gained greater than 6 lbs/year (Table 1). Weight change did not differ by gender.

**Table 1:** Distribution of weight change among adults ages 18-35 in an academic general internal medicine clinic

Weight change [n (%)]	Total (n = 365)	Men (n = 90)	Women (n = 275)	*P *value*
Weight loss	156 (43%)	37 (41%)	119 (43%)	0.57
0-2.9 lbs/year gain	119 (33%)	30 (33%)	89 (32%)	
3-5.9 lbs/year gain	56 (15%)	11 (12%)	45 (16%)	
6-11.9 lbs/year gain	23 (6%)	8 (9%)	15 (5%)	
>12 lbs/year gain	11 (3%)	4 (4%)	7 (3%)	

* *P *value was computed using Chi-square test to evaluate the association between weight change and gender.

Among individuals gaining more than 3 lbs/year, physicians documented weight gain in only 10% (9/90). Documentation was no higher among individuals gaining more than 6 lbs/year than among those gaining between 3-5.9 lbs/year (15% vs. 7%, p = 0.26). Of the nine patients with documented weight gain, three were entered as encounter diagnoses, and six were documented in the note text. None had weight gain added to an ongoing problem list.

Discussions related to weight were initiated by the patient in four cases, by the physician in three, and two were indeterminate. Physician counseling for the nine patients with documented weight gain included weight-specific counseling for three, general diet and exercise counseling for four, and no documented counseling for two (Table 2).

**Table 2:** Physician diet and exercise counseling by presence or absence of weight gain documentation

Counseling category [n (%)]	Total (n = 90)	Documentation of weight gain* (n = 9)	No documentation of weight gain (n = 81)	*P *value^†^
Weight specific counseling	5 (6%)	3 (33%)	2 (2%)	0.002
General diet and exercise counseling	32 (36%)	4 (44%)	28 (34%)	
No active counseling	53 (59%)	2 (22%)^‡^	51 (63%)	

* This category includes individuals with documentation of weight gain as an encounter diagnosis (n = 3) or documentation within the note text (n = 6).

^† ^*P *value was computed using Fisher's Exact test to evaluate the association between diagnosis of weight gain and counseling category.

^‡ ^Of note, the two individuals categorized in this cell had BMIs of 18-19 at the time of weight gain diagnosis.

Of the 81 individuals with no documented diagnosis of weight gain, 63% had no documented counseling, but 34% had documentation of general diet or exercise counseling. Patients for whom weight gain was documented were more likely to have diet and exercise counseling (p = 0.002) (Table 2).

Over a median of 388 days post-health maintenance visit, 8% of patients (10/126) became overweight, and none became obese.

## Discussion

To our knowledge this is the first study to evaluate physician documentation of and counseling for weight gain. Among this sample of normal weight young adults, almost a quarter gained more than 3 lbs/year. In the majority of cases, this weight gain was undocumented, and patients did not receive diet or exercise counseling. Even when weight gain was documented, it was not routinely added to the patient's ongoing problem list. Weight gain patterns observed among young adults in our study were similar to those seen in longer-term epidemiologic studies[9,10]. Rates of physician diagnosis and counseling for weight gain in our study also parallel national rates of physician documentation and counseling for overweight and obesity[11-14].

Epidemiologic studies show that many adults experience a sustained pattern of weight gain over time, thus underscoring the importance of prevention. Early detection of weight gain may be an effective method of targeting patients for early counseling for primary prevention of overweight. Although diet and exercise counseling is time-consuming,[15] our study showed that physicians are already providing general diet and exercise counseling to a third of their patients. Physicians could further tailor these ongoing discussions by using a patient's weight gain to direct a focused discussion on weight maintenance. Patients who are gaining weight may also be more motivated to make a change in their lifestyle due to a heightened risk perception[16]. However, because physicians rarely recognize weight gain among their patients, tools are needed to help identify individuals who are rapidly gaining weight. Point-of-care alerts for weight gain could remind physicians to respond during preventive visits and also prompt opportunistic counseling during urgent care visits. Use of graphical representations of weight over time may also highlight weight change as a concern for both the physician and patient. Further, in order to increase physician counseling rates, resources are also needed to facilitate brief, targeted counseling on diet, exercise and weight maintenance. More studies are needed to evaluate if early detection paired with brief counseling is effective in curbing patients' longer term weight trajectory. This study has several limitations. First, it was conducted at a single academic institution and had a higher than average proportion of patients with psychiatric diagnoses. As a result, the results may not be generalizable to all other practices. Second, we did not ascertain the etiology of patient weight gain (ie. medication use versus change in lifestyle behaviors). Third, defining rapid weight gain as greater than 3 lbs/year was necessarily arbitrary given that no prior research has established a meaningful cut-off value. Lastly, chart review is dependent on the accuracy of physician documentation. Although rates of counseling may be higher than documented, the intensity of undocumented counseling is likely to be low.

## Conclusions

Many young adults seen in the clinic setting have rapid weight gain that remains unrecognized. Methods to increase physician identification of weight gain are needed to create opportunities for focused weight-related preventive counseling at a time when patients may be motivated to make a lifestyle change.

## Competing interests

The authors declare that they have no competing interests.

## Authors' contributions

JWT participated in design of the study, conducted chart review, performed the statistical analyses, and drafted the manuscript. RK participated in the design of the study and contributed critical feedback with manuscript revision. JT was responsible for electronic acquisition of the data. DB participated in the design of the study, conducted statistical analyses, and contributed critical feedback with manuscript revision. All authors read and approved the final manuscript.

## Pre-publication history

The pre-publication history for this paper can be accessed here:

http://www.biomedcentral.com/1471-2296/11/31/prepub
